# Modulation of Methoxyfenozide Release from Lignin Nanoparticles Made of Lignin Grafted with PCL by ROP and Acylation Grafting Methods

**DOI:** 10.1021/acs.langmuir.3c03965

**Published:** 2024-03-01

**Authors:** Alvaro Garcia, Carlos E. Astete, Rafael Cueto, Cristina M. Sabliov

**Affiliations:** † Biological & Agricultural Engineering, Louisiana State University and LSU AgCenter, Baton Rouge, Louisiana 70803, United States; ‡ Department of Chemistry, Louisiana State University, Baton Rouge, Louisiana 70803, United States

## Abstract

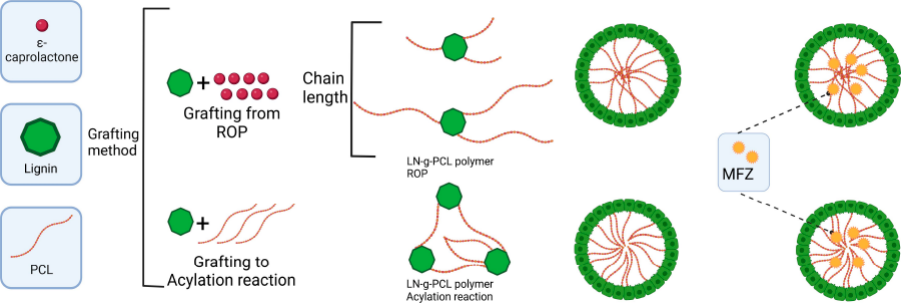

An efficient and sustainable agriculture calls for the development of novel agrochemical delivery systems able to release agrochemicals in a controlled manner. This study investigated the controlled release of the insecticide methoxyfenozide (MFZ) from lignin (LN) nanoparticles (LNPs). LN-grafted poly(ε-caprolactone) (LN-*g*-PCL) polymers were synthesized using two grafting methods, ring-opening polymerization (ROP)(LN-*g*-PCL_p_) and acylation reaction (LN-*g*-PCL_a_), creating polymers capable of self-assembling into nanoparticles of different properties, without surfactants. The LN-*g*-PCL_p_ polymers exhibited a degree of polymerization (DP) from 22 to 101, demonstrating enhanced thermal stability after LN incorporation. LNPs loaded with MFZ exhibited a spherical core–shell structure with a hydrophilic LN outer layer and hydrophobic PCL core, with sizes affected by grafting methods and DP. LNPs controlled MFZ release, displaying variation in release profiles depending on the grafting methodology used, LN-*g*-PCL_p_ DP, and temperature variations (23 to 30 °C). LNPs formulated with LN-*g*-PCL_a_ showed a cumulative release of MFZ of 36.78 ± 1.23% over 196 h. Comparatively, increasing the DP of the LN-*g*-PCL_p_ polymers, a reduction of the LNPs release rate from 92.39 ± 1.46% to 70.59 ± 2.40% was achieved within the same time frame. These findings contribute to identifying ways to modulate the controlled release of agrochemicals by incorporating them in renewable-based LNPs.

## Introduction

Plastics have been implemented worldwide across industrial sectors due to their versatility and low cost, increasing plastic production since the 1950s.^1^ While implementing plastics in agriculture increased productivity in many sectors, it also increased plastic waste generation and agricultural farmland contamination.^2,3^ Consequently, agricultural plastic waste raises concerns about microplastic contamination of soil ecosystems and the possible uptake by plants, spreading plastics within the food chain.^3^ Sustainable alternatives to plastics are needed to maintain or enhance agricultural productivity while minimizing plastic waste. In order to address this challenge, biopolymers, biofriendly materials with controlled degradation profiles, have emerged as a promising alternative to address the challenges of plastic waste.^4^

Natural biopolymers, such as lignin (LN), have a vast potential for applicability in synthesizing lignin-modified polymers. LN is of great interest given that it is one of the most abundant natural aromatic substances in nature, obtained from the pulp and paper industry as a byproduct.^5^ The abundance of hydroxyl groups in aromatic and aryl chains allows the functionalization of LN by covalently attaching synthetic polymers.^6^ Additionally, the modification of LN, a hydrophilic polymer, with hydrophobic polymers by grafting implements amphiphilicity to the resulting lignin-grafted polymer, allowing the polymer to assemble into nanostructures without the need for surfactants^7^ and to control the release of drugs from these nanostructures.^8,9^

A previous study employed lignin-based nanoparticles (LNPs) as a delivery system to control the release and enhance the translocation of methoxyfenozide (MFZ), a nonsystemic insecticide, in soybean plants.^10^ LNPs were synthesized from lignin-*graft*-poly(lactic-*co*-glycolic) acid (PLGA) and loaded with MFZ at 2.7% w/w, presenting a size of 113.8 ± 3.5 nm, a PDI of 0.313 ± 0.029, and a zeta potential of −53.3 ± 6.9 mV. The authors reported that LNPs facilitated the controlled release and translocation of MFZ through soybean tissues, making it an alternative delivery system in agricultural applications. Even though LN-modified PLGA nanoparticles exhibit a promising delivery system, the cost of the PLGA, which is ten times higher than that of other biodegradable polymers, poses a challenge. Poly(ε-caprolactone) (PCL), an FDA-approved polymer with hydrophobic, biodegradable, and nontoxic properties, exhibits a slow degradation rate compared with other biodegradable polymers, allowing the controlled release of entrapped compounds for extended periods.^11,12^

The significance of this work relies on the potential improvement in the control of agrochemical release and the reduction of environmental contamination of the delivery system synthesized from a renewable polymer. MFZ, an insecticide widely used to control lepidopteran larvae, was used as a model agrochemical. To the best of our knowledge, there are no published studies on LN-grafted PCL (LN-*g*-PCL) assembly into nanoparticles and their use as an agrochemical delivery system. To achieve this goal, we explored two grafting methods, ring-opening polymerization (ROP)(LN-*g*-PCL_p_) and acylation reaction (LN-*g*-PCL_a_), creating polymers capable of self-assembling into nanoparticles of different properties, without surfactants. Our hypothesis was that two distinct grafting methods and the manipulation of the PCL chain length grafted to LN will impact the controlled release of agrochemicals from LNPs, resulting in different release profiles. Herein, thermal properties and chemical structure analysis of polymers and the impact of grafting techniques, degree of polymerization (DP) of LN-*g*-PCL_p_, and temperature increase on LNPs release profile of MFZ are reported.

## Materials and Methods

### Materials

PCL (*M*_n_ = 45000 g mol^–1^), ε-caprolactone (CL, >99%), stannous 2-ethyl hexanoate (Sn(Oct)_2_, purity 92.5–100.0%), cyclohexanol (purity 98.5%), and 2-chloro-4,4,5,5-tetramethyl-1,3,2-dioxaphospholane (TMDP, purity 95%) were acquired from Sigma-Aldrich (St. Louis, MO). Alkaline LN was acquired from TCI Inc. (Portland, OR). Methanol (purity ≥99%), dichloromethane (DCM, purity ≥99.9% extra dry), oxalyl chloride (purity ≥99%), dimethylformamide (DMF, purity ≥99.5%), dimethyl sulfoxide (DMSO, purity ≥99.7%), ethyl ether (purity ≥99%), toluene (purity ≥99.5%, extra dry), and trehalose (purity ≥99%) were acquired from Fisher Scientific (Pittsburgh, PA).

### Synthesis of LN-*g*-PCL Polymers

**Scheme 1:**
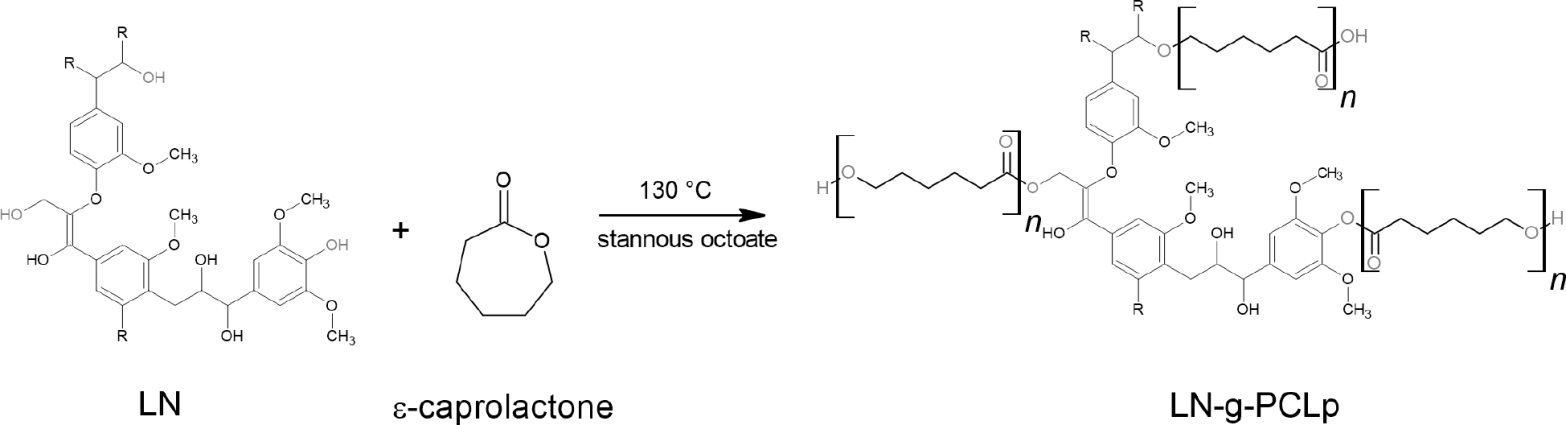
Synthesis of LN-*g*-PCL_p_ by the ROP Reaction

Synthesis 1: “Grafting from” LN-*g*-PCL_p_ polymers were synthesized by ROP of the monomer CL, following a slightly modified reported procedure,^13^ as described in Scheme 1. Alkali lignin was dried in a vacuum oven before the synthesis. The synthesis was conducted under anhydrous bulk conditions without any solvent under argon flux. Briefly, 2 g of alkali lignin was placed in a 250 mL 3-neck round-bottom flask, followed by adding CL at a given ratio. Different CL/LN ratios were used (w/w) = 2, 6, and 10 to graft LN. Therefore, three different DPs were obtained (22, 57, and 101 by ^1^H NMR) with different equivalent lengths of the PCL chain.

The mixture remained under consistent magnetic agitation at ambient temperature until total dissolution of LN was achieved. Then, the mixture was heated and stirred in an oil bath at 130 °C. Subsequently, 0.2% (v/v according to CL volume) of Sn(Oct)_2_ was added as a catalyst. The grafting reaction proceeded for 24 h with constant stirring at 200 rpm under argon flow to create anhydrous conditions. When the reaction was complete, the solution was precipitated dropwise into 200 mL of cold methanol. The supernatant was discharged, and the precipitate was washed five times with cold methanol.

Next, the resulting grafted polymer was diluted in 200 mL of DCM and washed five times with distilled water to remove unreacted LN. Then, the polymer was concentrated in a Rotavapor R-300 (Buchi Corporation, New Castle, DE). The resulting solids were frozen at −80 °C for 24 h and lyophilized using a freeze-dryer (FreeZone Plus 2.5; Labconco, Kansas City, MO) to remove all the water and solvents from the polymer. Finally, the LN-*g*-PCL_p_ polymer was stored in a desiccator at room temperature for future use.

**Scheme 2:**
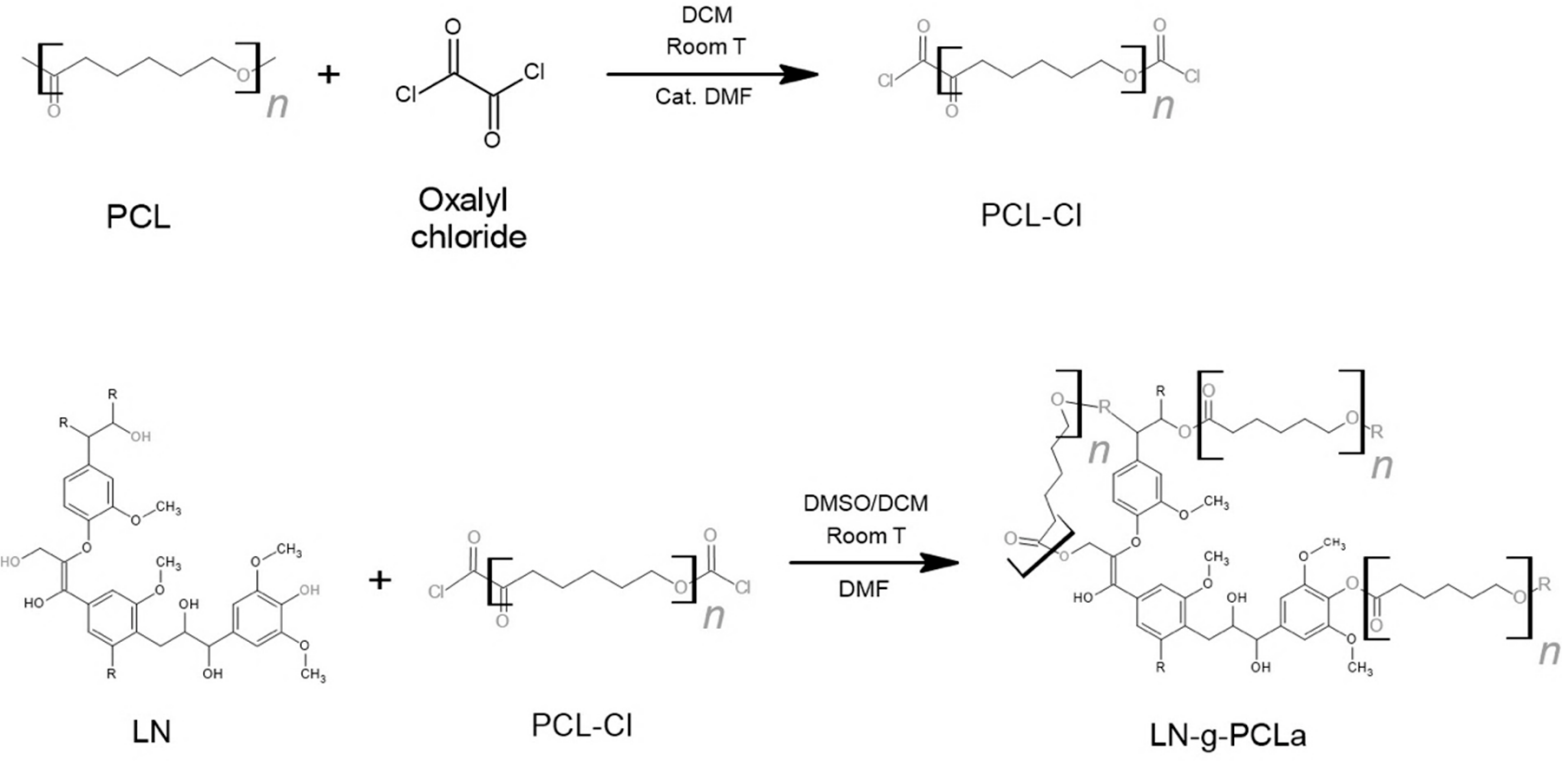
Synthesis of LN-*g*-PCL_a_ by the Acylation Reaction R are the randomly distributed monolignols of lignin (*p*-coumaryl, sinapyl, and coniferyl alcohol).

Synthesis 2: “Grafting to” LN-*g*-PCL_a_ polymers were synthesized by the acylation reaction, following a modified reported method.^7^Scheme 2 shows the acylation reaction procedure. Briefly, 4 g of PCL (103 DP by ^1^H NMR) was dissolved in 50 mL of DCM at room temperature in a three-neck round-bottom flask under anhydrous conditions with argon flux. Oxalyl chloride (110 μL) was added dropwise using a glass syringe, and 4 mL of DMF was added as a catalyst. The reaction was conducted at room temperature at 300 rpm for 4 h.

DCM was evaporated in a Buchi R-300 rotavapor (Buchi Corporation, New Castle, DE) to obtain a viscous solution of PCL-Cl. The coupling reaction involved dissolving PCL-Cl in 50 mL of DCM, which was added dropwise to 2 g of alkali lignin dissolved in 50 mL of DMSO. Then, the mixing was left to react for 24 h under argon flow. At the end of the reaction, ethyl ether (200 mL) was added to the mixture to precipitate the LN-*g*-PCL_a_ polymer.

Then, the precipitation, washing, and lyophilization were done following the same methodology stated in the “Synthesis 1” section, with the final polymer named LN-*g*-PCL_a_ for future experiments. For the purpose of being consistent, we included the DP of PCL for all polymers synthesized by acylation and polymerization. Hence, a value of 103 DP was included to indicate the DP of PCL grafted to LN by acylation to form LN-*g*-PCL_a_. The DP value was determined by ^1^H NMR analysis of commercial PCL.

### Characterization of LN-*g*-PCL Polymers

Fourier transform infrared (FTIR) spectroscopic analysis was used to confirm the attachment of LN to PCL using a Bruker Tensor 27 instrument (Bruker 500, Billerica, MA). Dried samples were analyzed using 32 scans, at a resolution of 4 cm^–1^, between the range of 400 and 600 cm^–1^. Changes in hydroxyl and carbonyl groups were analyzed to evaluate the interaction between polymers.^13^

Nuclear magnetic resonance (NMR) spectra were recorded on a Bruker 400 (Billerica, MA) instrument at 400 Hz in deuterated chloroform (CDCl_3_). ^1^H NMR spectra allowed the estimation of the average length of the PCL arms grafted onto LN by calculating the ratio of integrals between the areas of the signal peak at 4.03 ppm (repeating −CH_2_O−) and 3.65 ppm (terminal −CH_2_OH).^14,15^

Quantitative ^31^P NMR was used to determine the hydroxyl content of lignin following a slightly modified previously reported method.^16^ Briefly, 20 mg of dried LN were dissolved in a mixture of 0.5 mL of pyridine/deuterated chloroform (1.6:1, v/v). Cyclohexanol (100 μL/mL) was used as an internal standard, and TMDP (0.1 mL) was added to phosphitylate the hydroxyl groups. ^31^P NMR spectra acquisition was performed after 15 min of the addition of TMDP. The spectra were acquired using an inverse-gated decoupling pulse with 12 s relaxation delay with 256 scans. The chemical shift was calibrated by the peak of the product of TMDP plus water at 132.2. The hydroxyl content of lignin and LN-*g*-PCL polymers was determined based on the concentration of cyclohexanol in the internal standard.

Monomer conversion was calculated by using the integral peaks of the ^1^H NMR spectra as follows:
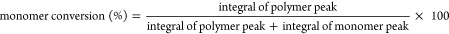
1The polymerization yield was calculated using the equation
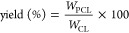
2where *W*_CL_ is the initial weight of CL and *W*_PCL_ is the final weight of PCL in the grafted polymer.

Differential scanning calorimetry (DSC) was performed using a TA Q100 DSC instrument (TA Instruments, New Castle, DE). The crystallization (*T*_c_) and melting (*T*_m_) temperatures were determined using a method reported elsewhere.^15^ About 2–4 mg of pure LN, PCL, and LN-*g*-PCL polymer samples were sealed in an aluminum pan, followed by a heating and cooling cycle from 0 to 120 °C at 10 °C/min rate under a nitrogen atmosphere.

The crystallinity of the samples was calculated according to the equation
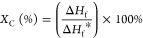
3where Δ*H*_f_ refers to the melting enthalpy (J g^–1^) obtained from the fusion peak of DSC and Δ*H*_f_* = 136.1 J g^–1^, which is the heat of fusion for 100% crystalline PCL.^14^

Thermogravimetric analysis (TGA) was used to evaluate the thermal stability of pure LN, PCL, and LN-*g*-PCL polymer samples on a TA TGA550 (TA Instruments). About 3–5 mg of the samples were placed in an aluminum pan and were heated to 600 °C at a heating rate of 50 °C min^–1^ under a nitrogen flow rate.

Gel permeation chromatography (GPC) was used to determine the molecular weight (*M*_w_) and number-average molecular weight (*M*_n_) of PCL and LN-*g*-PCL polymers. The polydispersity index (PDI) was defined as the ration of *M*_w_ to *M*_n_. 5 mg of LN-*g*-PCL_p_ polymers or PCL was dissolved overnight in 1 mL of THF and then filtered using a 0.45 μm PTFE syringe filter. The *M*_w_ and *M*_n_ analysis for LN-*g*-PCL_p_ polymers was performed on newly synthesized polymers with DP similar to those listed in Table 1. Specifically, polymers with DPs of 20, 53, and 99 were analyzed in comparison to the DPs of 22, 57, and 101 reported in Table 1. An EcoSEC Elite GPC system was used for separation equipped with two TSK gel columns from Tosoh (4.6 mm × 15 cm × 3 μm, Super HZ2400, Super HZ4000) and a guard column (Super HZ-L, 3 μm × 4.6 mm × 2 cm, Tosoh Bioscience LLC, King of Prussia, PA). Two detectors were used for detection: the EcoSEC differential refractive index detector and the Lens3 multiangle light scattering detector (Tosoh) equipped with a 505 nm diode laser at 20 mW. 20 μL of sample was injected using THF stabilized with 250 ppm of BHT at 0.3 mL/min. The EcoSEC was calibrated with polystyrene standards or multiangle light scattering. The polymers mass was determined using a refractive index (d*n*/d*c*) of 0.071 mL/g for all the samples containing PCL.^17^

**Table 1:** Properties of LN-*g*-PCL Copolymers

	*X*_c_ (%)^a^	OH (mmol/g)^b^	*M*_n_ (Da)^c^	*M*_w_ (Da)^c^	PDI^c^	monomer conversion (%)^d^	polymerization yield (%)
LN		3.94					
LN-*g*-PCL_p_22DP	59.4 ± 3.3	0.75	7199	7522	1.04	96	91.1
LN-*g*-PCL_p_57DP	53.0 ± 2.6	0.39	17777	20812	1.17	98	66.7
LN-*g*-PCL_p_101DP	49.0 ± 2.2	0.17	21943	31817	1.45	99	89.5
LN-*g*-PCL_a_103 DP	31.0 ± 1.2	1.13					
PCL	48.9 ± 2.1	0.10	30195	36225	1.2		

a *X*_c_ determined by DCS.

b OH quantification obtained by ^31^P NMR.

c Data obtained by GPC.

d Data obtained by ^1^H NMR.

### LN-*g*-PCL NPs Synthesis

LNPs were synthesized by the oil-in-water (O/W) emulsion evaporation technique following the methodology previously reported^7,10^ using the LN-*g*-PCL polymers. This emulsion technique forms a core–shell structure with a hydrophilic LN outer layer and a hydrophobic PCL core. Briefly, lignin-*g*-PCL (400 mg) was dissolved in DCM (20 mL). Deionized water (120 mL), as aqueous phase, was employed while the organic solution was gently introduced in droplets. Subsequent to this, microfluidization was performed in four cycles utilizing an M-110P microfluidizer (Microfluidics, Newton, MA). The organic solvent was subjected to evaporation using a Rotavapor R-300 apparatus (Buchi Corporation, New Castle, DE). Introducing trehalose (800 mg) as a cryoprotectant, the solution was then frozen at −80 °C for a duration of 24 h, and the subsequent lyophilization was conducted employing a FreeZone Plus 2.5 freeze-dryer (Labconco, Kansas City, MO). Agrochemical loading was accomplished following the same method, with the exception that methoxyfenozide (40 mg) was added in the organic phase.

### Nanoparticle Characterization

Size distribution, polydispersity, and zeta potential of the fresh LN-*g*-PCL nanoparticles were determined by dynamic light scattering (DLS) using a Malvern zetasizer ZS (Malvern, Panalytical, Westborough, MA) at 25 °C. Briefly, nanoparticles were resuspended in water at a concentration of 0.2–0.5 mg/mL and placed in a cuvette for characterization.

The morphology and aggregation of resuspended nanoparticles were analyzed by transmission electron microscopy (TEM) using a JEM-1400 TEM (Jeol USA Inc., Peabody, MA). LN-*g*-PCL nanoparticles resuspended in water were placed onto a copper grid with a contrast agent (uranyl acetate, 1%) and dried at room temperature. Then, the grid was placed in a TEM chamber for further analysis.

Resuspended freeze-dried LN-*g*-PCL nanoparticles were utilized for evaluating the loading capacity (LC) and entrapment efficiency (EE). 20 mg of nanoparticle powder was suspended in 2 mL of water. Subsequently, 100 μL of the mixture was diluted in 500 μL of acetonitrile. The total agrochemical content within the solution was quantified using high-performance liquid chromatography (HPLC) set to a wavelength of 254 nm by injecting 20 μL of the sample. The Agilent HPLC system used was equipped with an LC-20AT pump and an SPD-20A PDA detector and SIL-20AC interfaced with the LC- solution software system (Agilent, USA). An elution gradient was used by using water solvent A and acetonitrile as solvent B. The initial condition was set to 30% solvent B for 10 min. Subsequently, a solvent gradient was set to 90% solvent B within 10 min, and the gradient was held for 5 min. A flow rate of 1 mL min^–1^ was used with the Zorbax Agilent C18 column (150 mm × 4.6 mm i.d. × 5 μm particle size). The calculation of LC and EE for the nanoparticles was accomplished via the two following equations.
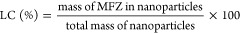
4
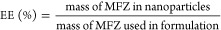
5The number of particles from LN-*g*-PCL copolymers was measured via nanoparticle tracking analysis (NTA) using a ZetaView Quatt PMX-420 instrument (Particle Metrix GmbH, Meerbusch, Germany). Resuspended LN-*g*-PCL nanoparticles were tracked using a laser (λ = 488 nm) and scattered light.

### Agrochemical Release

The release of MFZ was performed by the dialysis method, and the agrochemical concentrations were measured by high-performance liquid chromatography (HPLC). Briefly, LNPs were dispersed in water at a concentration of 10 mg/mL of NPs and placed in a dialysis bag (MWCO of 12–14 kDa, Fisherbrand, USA) and immersed in 1 L of PBS solution at 23 and 30 °C. 300 μL aliquots were taken at 0, 1, 2, 3, 6, 12, 24, 48, 72, 96, 144, and 192 h with a replacement of the PBS every 24 h. Extraction of MFZ from the nanoparticles was performed by adding 100 μL of sample to 500 μL of acetonitrile. For each aliquot, agrochemical concentrations were quantified by using HPLC analysis, as mentioned above. For the cumulative release analysis, the MFZ amount at time zero was considered as the initial 100% for each NPs system, and the release was reported relative to this initial percentage.

### Statistical Analysis

Experimental data were collected in triplicate (*n* = 3) and analyzed using R Studio for Windows v2023.06.0 + 421 (RStudio Inc., Boston, MA). The *p* value was defined as *p* < 0.05 using one-way ANOVA to compare statistical differences between treatments and two-way ANOVA to determined significant differences between treatments and within treatment though the release time frame. Tukey HSD was performed to test differences among sample means (*p* < 0.05). The data are reported as the mean ± standard deviation.

## Results and Discussion

Biopolymers have emerged as an alternative in the pursuit of eco-friendly materials that can control the release of agrochemicals for sustainable agriculture. Lignin-modified polymers have the potential to be used in applications in various fields, including biomedicine, packaging, and agriculture.^18,19^

In this study, we used two approaches for the modification of LN to synthesize LN-*g*-PCL copolymers, exploring the “grafting from” ring-opening polymerization and “grafting to” acylation reaction technique. Subsequently, we developed nanodelivery systems for the controlled release of MFZ. The different polymeric structures formed by the two grafting methodologies, alongside the controlled manipulation of the DP provided by the ROP method, facilitated the controlled release of MFZ.

### Polymer Characterization

**Figure 1:**
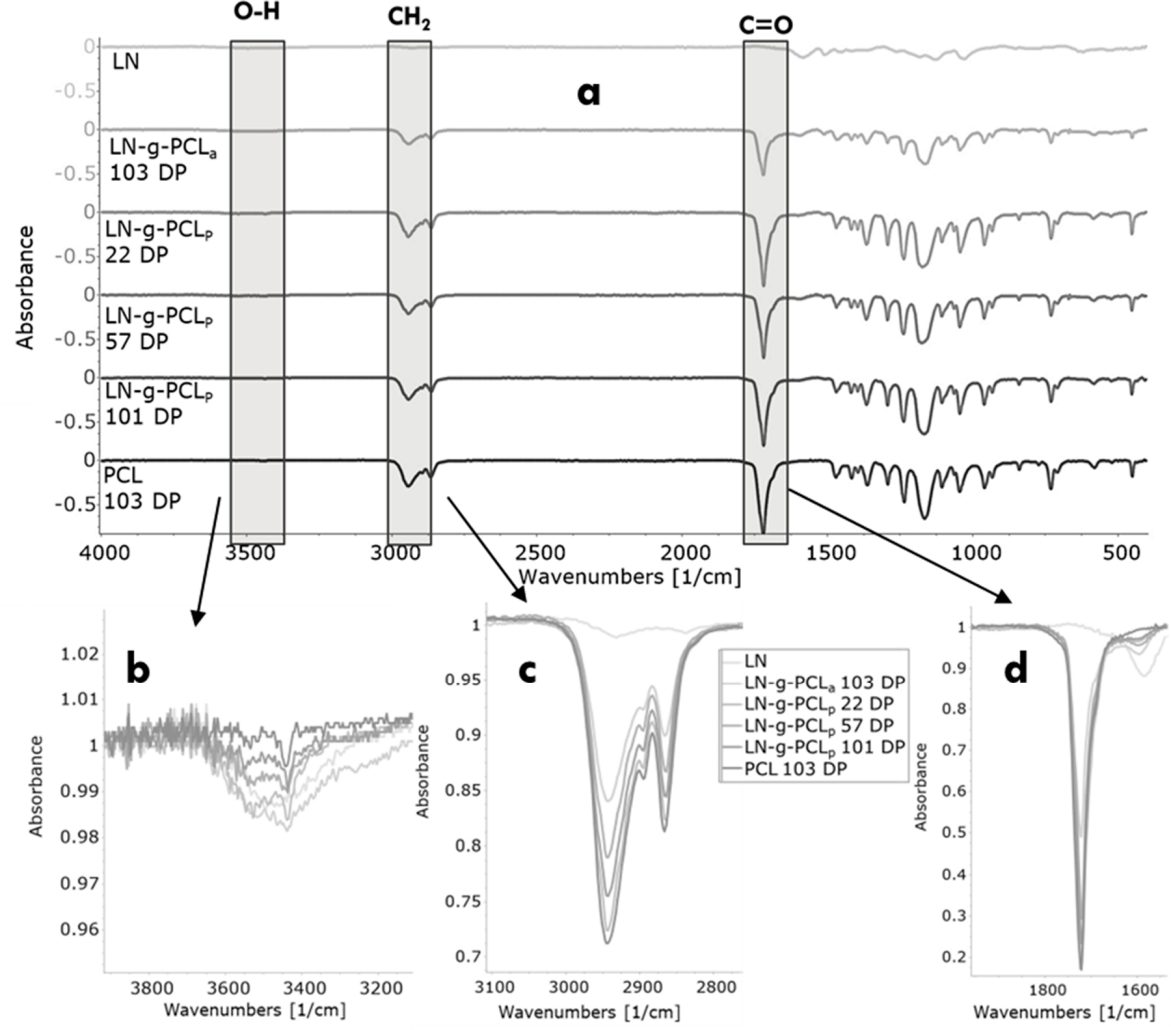
FT-IR analysis for LN, LN-*g*-PCL_p_ (22, 57, and 101 DP), LN-*g*-PCL_a_ (103 DP), and PCL (103 DP) whole spectra (a), OH region (b), CH_2_ region (c), and C=O region (d).

Inter- and intramolecular interactions between LN and PCL can be observed in the FT-IR spectra (Figure 1a), confirming PCL attachment to LN. Functional groups such as methylene hydroxyl (OH, 3000–3700 cm^–1^_,_Figure 1b), (CH_2_, 2945–2864 cm^–1^, Figure 1c), carbonyl (C=O, ∼1721 cm^–1^, Figure 1d), and aromatic ring (1600 cm^–1^) were observed in the FTIR spectra. The shifting intensity of the OH decreased in the copolymers LN-*g*-PCL after grafting compared to unmodified LN, indicating a reaction between the OH of LN and PCL during the grafting process due ester linkage formation.^20,21^ The strong C=O absorption peak for both PCL and LN-*g*-PCL copolymers suggests the formation of intermolecular hydrogen bond between the carboxylic group of PCL.^13,20^ Additionally, the presence of the aromatic ring, a characteristic of LN, confirms the incorporation of LN into the LN-*g*-PCL copolymer.^13,22^

**Figure 2:**
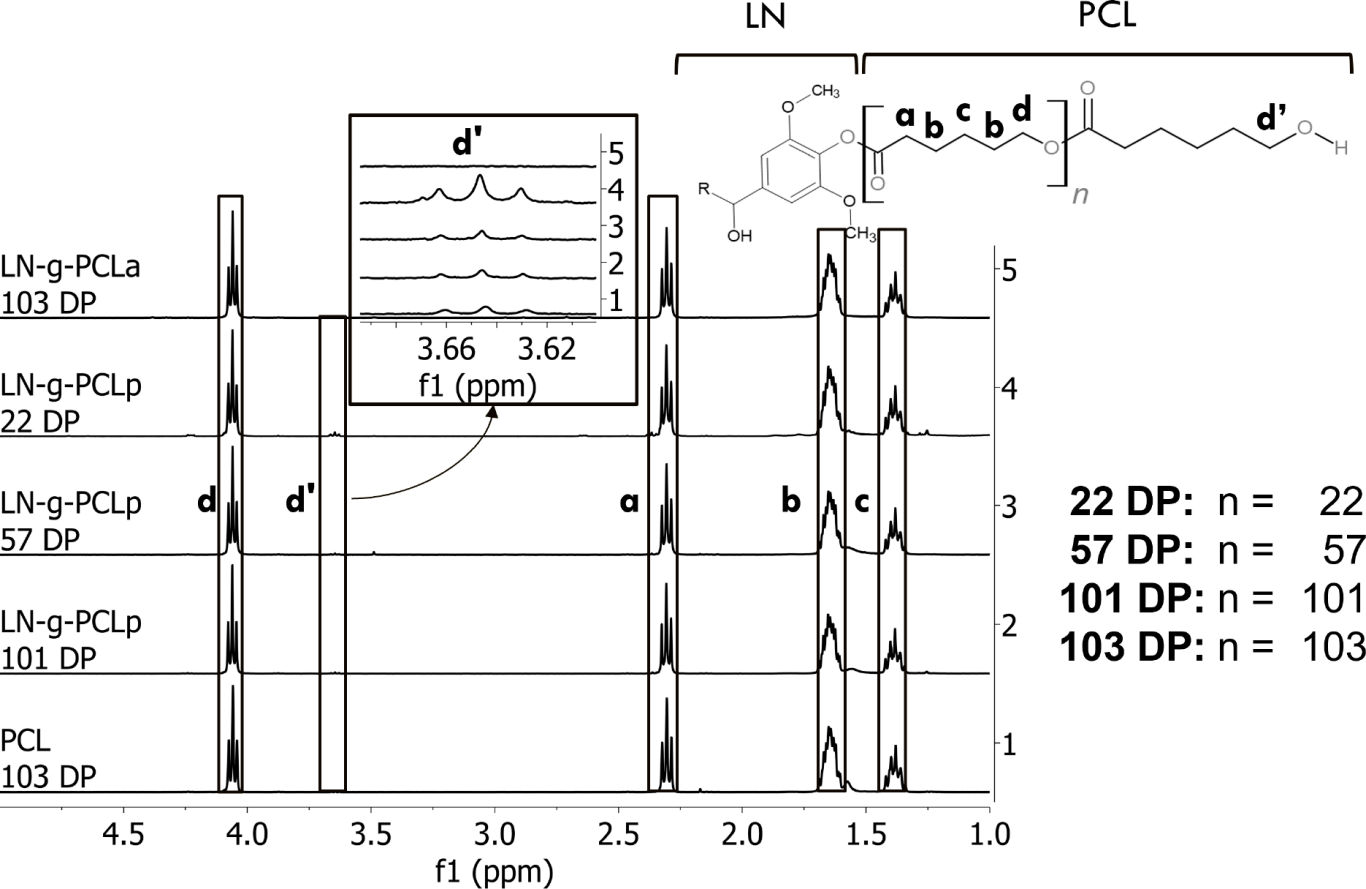
^1^H NMR spectra for LN-*g*-PCL_p_ (22, 57, and 101 DP), LN-*g*-PCL_a_ (103 DP) copolymers, and PCL (103 DP).

The chemical structure of LN-*g*-PCL was analyzed by ^1^H NMR and compared to PCL (Figure 2). Several PCL peaks were detected: 4.03 ppm (d, repeating −CH_2_O−), 3.65 ppm (d′, terminal −CH_2_OH), 2.29 ppm (a, −COCH_2_−), 1.63 ppm (b, −CH_2_−), and 1.38 ppm (c, −CH_2_−).^14^

The number of repeated units of CL grafted to LN, also known as DP, was calculated based on the integrative ratio of methylene subtypes, repeating −CH_2_O– (d) and terminal −CH_2_OH (d′).^14,15^ While the DP for ROP method copolymers ranged from 22 to 101, the LN-*g*-PCL_a_ copolymers synthesized through acylation reaction had no detectable terminal methylene peak, indicating complete modification of the end groups of the PCL chain (Figure 2). Additionally, the monomer conversion analysis shown in Table 1 reveals a high monomer conversion during bulk polymerization of LN and CL, as reported in previous studies.^23^

**Figure 3:**
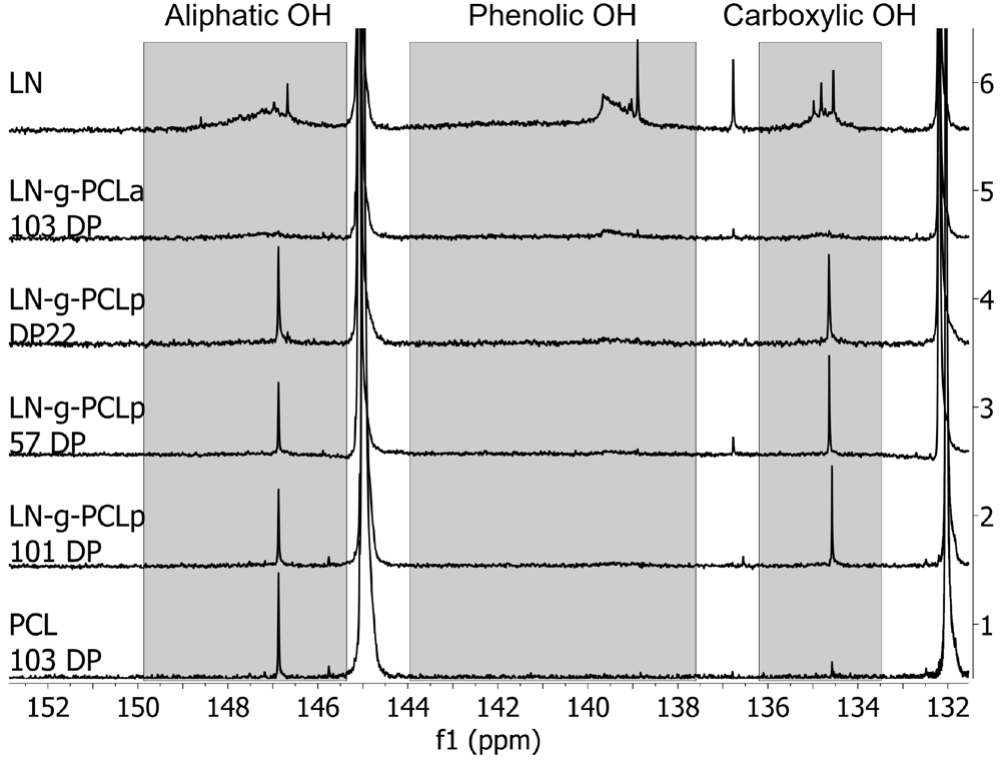
^31^P NMR spectra for LN, LN-*g*-PCL_p_ (22, 57, and 101 DP), LN-*g*-PCL_a_ (103 DP), and PCL (103 DP).

Furthermore, we explored the changes in the LN OH after the modification with PCL, analyzing reductions in aliphatic, phenolic, and carboxylic OH by grafting techniques using ^31^P NMR. Aliphatic (145.0–149.0 ppm), phenolic (140.4–144.4 ppm), and carboxylic OH (134–136 ppm) can be identified in Figure 3. Quantitative analysis showed an average reduction for all copolymers of 85.86 ± 6.35% in the aliphatic OH and 78.31 ± 22.88% in phenolic OH after modification of LN. Moreover, results in Table 1 show the total reduction of OH groups after modification of lignin. Increasing the ratio between CL and LN revealed a reduction in the total OH of LN. This trend is related to the miscibility of LN in CL during the bulk reaction, where a higher volume of CL increases the dissolution of LN, increasing the availability of OH for the ROP reaction. A similar effect was observed in the acylation reaction, where the reduced miscibility of LN and modified PCL in DCM:DMSO solution decreased the availability of OH of lignin, resulting in a higher content of OH in LN at the end of the reaction.

Notably, LN-*g*-PCL_a_ copolymers synthesized through acylation reaction exhibited an absence of aliphatic and carboxylic OH peaks of PCL, indicating the complete modification of OH of PCL with oxalyl chloride during the acylation reaction.^7^

While the precise quantification of aliphatic and carboxylic OH quantification was obstructed by the OH peaks generated during the ROP, our results confirmed that the polymerization of CL also started at the carboxylic OH of LN, leading to the PCL arm terminating with a carboxylic OH group.^13^ Overall, the grafting modifications resulted in different PCL chain attachments to LN, suggesting that the LN-*g*-PCL properties can be controlled depending on the grafting method employed.

**Figure 4:**
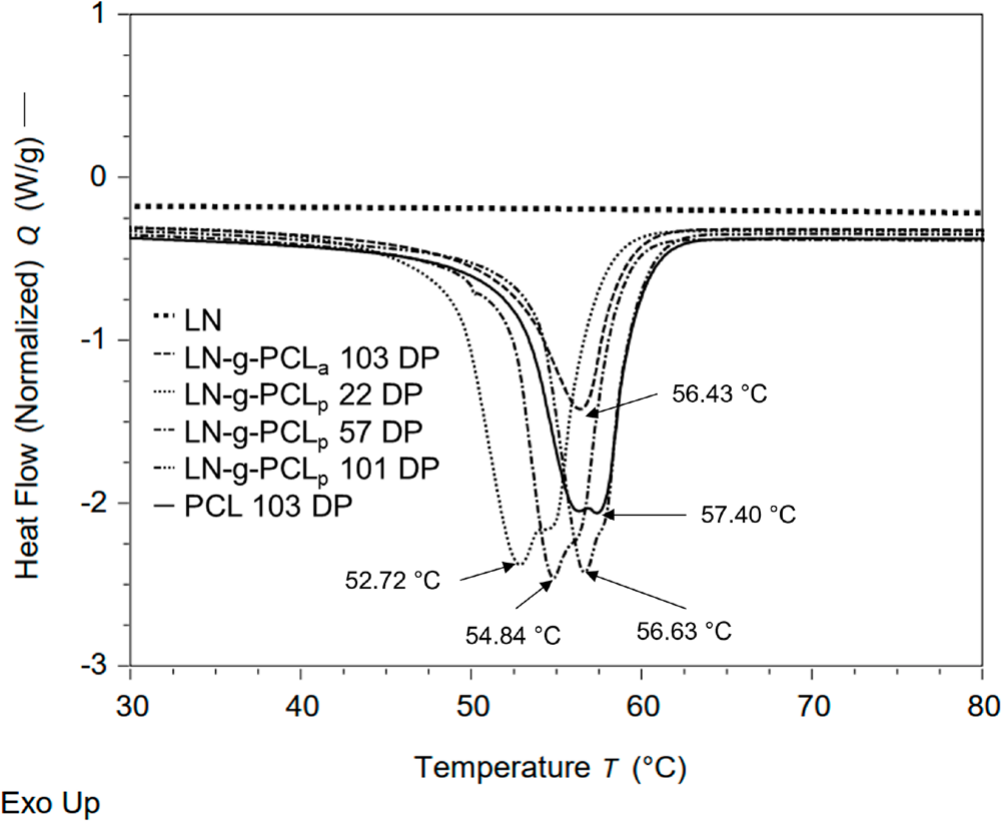
DSC analysis showing melting points (°C) for LN, LN-*g*-PCL_p_ (22, 57, and 101 DP), LN-*g*-PCL_a_ (103 DP), and PCL (103 DP).

DSC was employed to investigate the thermal characteristics of LN-*g*-PCL samples, alkaline LN, and PCL (Figure 4). In the temperature range of 0 to 120 °C, no glass transition temperature (*T*_g_) was observed for either the LN-*g*-PCL polymer samples or alkaline LN. However, melting and crystallization temperatures were detected for grafted polymers and PCL. Notably, the average melting point temperature (*T*_m_) of all LN-*g*-PCL polymers was 55.17 ± 1.81 °C, similar to the *T*_m_ of PCL (57.40 ± 1.32 °C). A trend emerged, showing that as the length of PCL arms increased, the *T*_m_ of the copolymers approached the *T*_m_ of PCL.^13,15,20,24^

Crystallinity, as measured by DSC, displayed a slight decrease with an increase in the DP of PCL attached to LN. Previous studies either reported an increase in crystallinity with DP^20,22^ or no trend.^13,14^ In this study, when the DP of the PCL arm was short (22 DP), the LN-*g*-PCL_p_ copolymer synthesized by ROP exhibited high crystallinity (59%). With an increase in the DP (101 DP), the crystallinity decreased to 48.96%. Moreover, the lowest crystallinity observed in this study (30.95%) was exhibited by the LN-*g*-PCL_a_ copolymer grafted by the acylation reaction.

The results demonstrated that the grafting method (ROP or acylation) and DP of the LN-*g*-PCL_p_ polymers influenced the copolymers’ crystallinity. Additionally, using a different method of grafting, such as the acylation reaction, resulted in PCL chains attached to the LN from both endings of PCL, leading to more rigid and compact chains and reduced crystallinity.

This behavior can be attributed to the nature of LN, a nonuniform and amorphous polymer, impeding the alignment of PCL arms and to the relaxation dynamics of grafted copolymers.^25^ Short PCL chain lengths exhibit a diffusive motion, allowing free movement and crystal formation. Conversely, long PCL chains length present a subdiffusive motion where the movement of the polymer chains is more restricted and slower due to interaction between PCL chains, resulting in lower crystallization.

**Figure 5:**
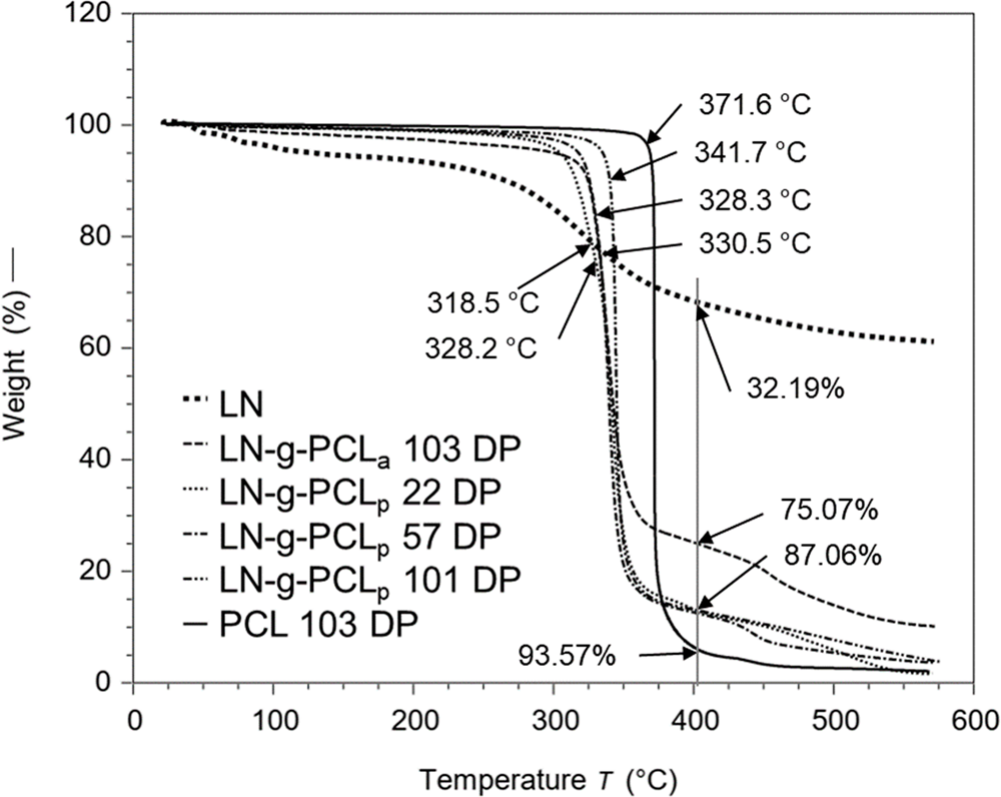
TGA analysis showing onset degradation temperature points (°C) and weight percentage loss (%) at 400 °C for LN, LN-*g*-PCL_p_ (22, 57, and 101 DP), LN-*g*-PCL_a_ (103 DP), and PCL (103 DP).

TGA was employed to investigate how the grafting method and DP influenced the thermal stability of the synthesized polymers. Typically, pure PCL undergoes a two-stage degradation process. In the initial phase, water, CO_2_, and carboxylic acid are generated as the released substances after a pyrolysis phase characterized by chain breakages and depolymerization processes.^26^ The results in Figure 5 indicate that the thermal stability of LN-*g*-PCL polymers is enhanced compared to neat PCL. The enhanced thermal stability can be attributed to the decomposition of LN aromatic compounds at temperatures higher than 600 °C.^27^

In this study, the total weight percent loss for PCL and LN at 400 °C was 93.57% and 32.19%, respectively, while the LN-*g*-PCL_p_ copolymer with 101 DP presented only 87.06%. Additionally, all other LN-*g*-PCL polymers with different DP showed similar total weight percent loss, regardless of the DP. In contrast, polymers grafted by the acylation reaction obtained the lowest total weight percent loss. Among all LN-*g*-PCL copolymers, the maximum rate of degradation temperature (MRDT) was similar (339.86 ± 1.30 °C), except for LN-*g*-PCL 101 DP, which presented a slightly higher MRDT of 344.36 °C, which is relatively lower than that of PCL (372.12 °C).

Conversely, the DP influenced the initiation temperatures of degradation.^20,24^ The initial decomposition temperature decreased from 341 to 328 °C when DP decreased from 101 to 22. In this case, the highest and lowest onset temperatures were found for PCL (371.63 °C) and LN (318.47 °C), respectively. These results suggest that grafting PCL onto LN enhances thermal stability and resistance,^14,20^ but the grafting method does not significantly alter the degradation pattern of copolymers.

### Nanoparticle Characterization

In the emulsion evaporation technique used for nanoparticle synthesis, the solvation of LN-*g*-PCL decreases during the evaporation of DCM. Consequently, the introduction of a LN-*g*-PCL/DCM solution into water leads to the aggregation of the polymer molecules and the formation of nanoparticles. The hydrophobic PCL segments avoid contact with the aqueous environment and aggregate to form the core of the nanoparticle, facilitating the encapsulation of the hydrophobic substances. Conversely, the hydrophilic LN segment, more compatible with the aqueous environment, orients toward the outer part of the nanoparticle. This LN shell enhances the stability of the NPs in an aqueous medium. Overall, this core–shell structure follows the typical behavior of amphiphilic copolymers in aqueous environments.

DLS was employed to determine the mean size, distribution, and zeta potential (Table 2) of LNPs synthesized from the four polymers. Notably, the grafting method affected the particle size, with LNPs from polymers synthesized by the acylation reaction showing the smallest size. The higher DP of polymers synthesized by ROP led to a larger particle size. This observation aligns with the behavior of amphiphilic copolymer micelles, where polymers with shorter hydrophobic sections resulted in smaller micelles.^28^

**Table 2:** Properties of LN-*g*-PCL Copolymers Nanoparticles*

	size (nm)	PDI	zeta potential (mV)	EE (%)	LC (%)	number of particles
LN-*g*-PCL_p_22DP	184.6 ± 3.5^b^	0.045 ± 0.01	–57.0 ± 1.8	77.8 ± 1.5^a^	7.8 ± 0.1^a^	2.70 × 10^10^
LN-*g*-PCL_p_57DP	269.6 ± 0.4^a^	0.099 ± 0.02	–58.8 ± 1.0	79.5 ± 1.5^a^	8.0 ± 0.1^a^	1.81 × 10^10^
LN-*g*-PCL_p_101DP	245.0 ± 1.5^a^	0.056 ± 0.02	–58.3 ± 1.2	78.9 ± 1.2^a^	7.9 ± 0.1^a^	1.85 × 10^10^
LN-*g*-PCL_a_103 DP	80.21 ± 0.2^c^	0.050 ± 0.02	–62.3 ± 6.6	57.6 ± 1.8^b^	5.8 ± 0.2^b^	2.73 × 10^10^

* EE: encapsulation efficiency; LC: loading capacity.

Results from this study showed that the LNPs’ mean size was influenced by the type of copolymers used. Despite the tendency for NPs made with amphiphilic copolymers to exhibit small sizes and narrow size distribution, LN-*g*-PCL_p_ NPs in this study presented a large mean size, considerably when DP was increased. The increase in LNPs’ mean size can be attributed to two factors: (1) limited solubility of LN-*g*-PCL_p_ polymers with higher DP during the nanoparticle synthesis process, promoting agglomeration of polymer chains, and (2) unrestrictive arrangement of PCL chains grafted to LN by ROP, creating a larger hydrophobic core within LNPs.^28^ Additionally, distinct molecular structures of the LN-*g*-PCL_a_ (103 DP) and LN-*g*-PCL_p_ (101 DP), even if of a similar DP, can influence LNPs’ size. In LN-*g*-PCL_a_, some PCL is attached at both ends, resulting in a more rigid but shorter PCL chain structure. Conversely, in LN-*g*-PCL_p_, PCL is grafted from one side, leading to a longer chain for the same DP. While an increase in DP from 22 to 57 leads to an increase in NPs size, no significant size differences were observed between 57 and 101 DPs. This can be attributed to changes in the *M*_w_ of polymers and the possible entanglement between PCL chains at higher DP. The increase of DP of PCL in LN-*g*-PCL_p_ polymers allows interaction between PCL chains, resulting in a more entangled core with no increase in the spatial properties of the hydrophobic core and NPs size.^29,30^ These differences in structure were evident in the crystallinity analysis included in Table 1, and they suggest that higher DP could reduce the diffusion of hydrophobic drugs through the more compact PCL matrix.^31^

Moreover, nanoparticle tracking analysis revealed a trend where lower NPs’ size resulted in higher number of NPs in the aqueous solution, and all LNPs were monodispersed and had a consistent negative zeta potential, with an average value ranging from −62.3 ± 6.6 to −57.0 ± 1.8 mV when suspended in water. This outcome is attributed to the various functional groups, such as phenolic and carboxylic OH, and possibly other anionic moieties present in LN forming the outer shell of the nanoparticles. These functional groups can become ionized in an aqueous environment, providing the anionic charge of the nanoparticles.^32^

This hypothesis is supported by ^31^P NMR analysis which showed a reduction but not complete conversion of aliphatic and phenolic groups in modified LN. In particular, there is a remaining 15.49 ± 10.73% of OH on average for all LN-*g*-PCL copolymers. This indicates the presence of unmodified OH groups that could contribute to the negative zeta potential. With regard to LN-*g*-PCL_a_ particles, the absence of a carboxylic OH peak in the ^31^P NMR analysis refers to the complete modification of the PCL end groups. However, the LN in LN-*g*-PCL_a_ still conserves some carboxylic and phenolic OH groups after grafting, contributing to the negative *Z* potential (Table 1). Hence, the results confirmed the influence of lignin’s functional groups on the negative *Z* potential of the nanoparticles.

**Figure 6:**
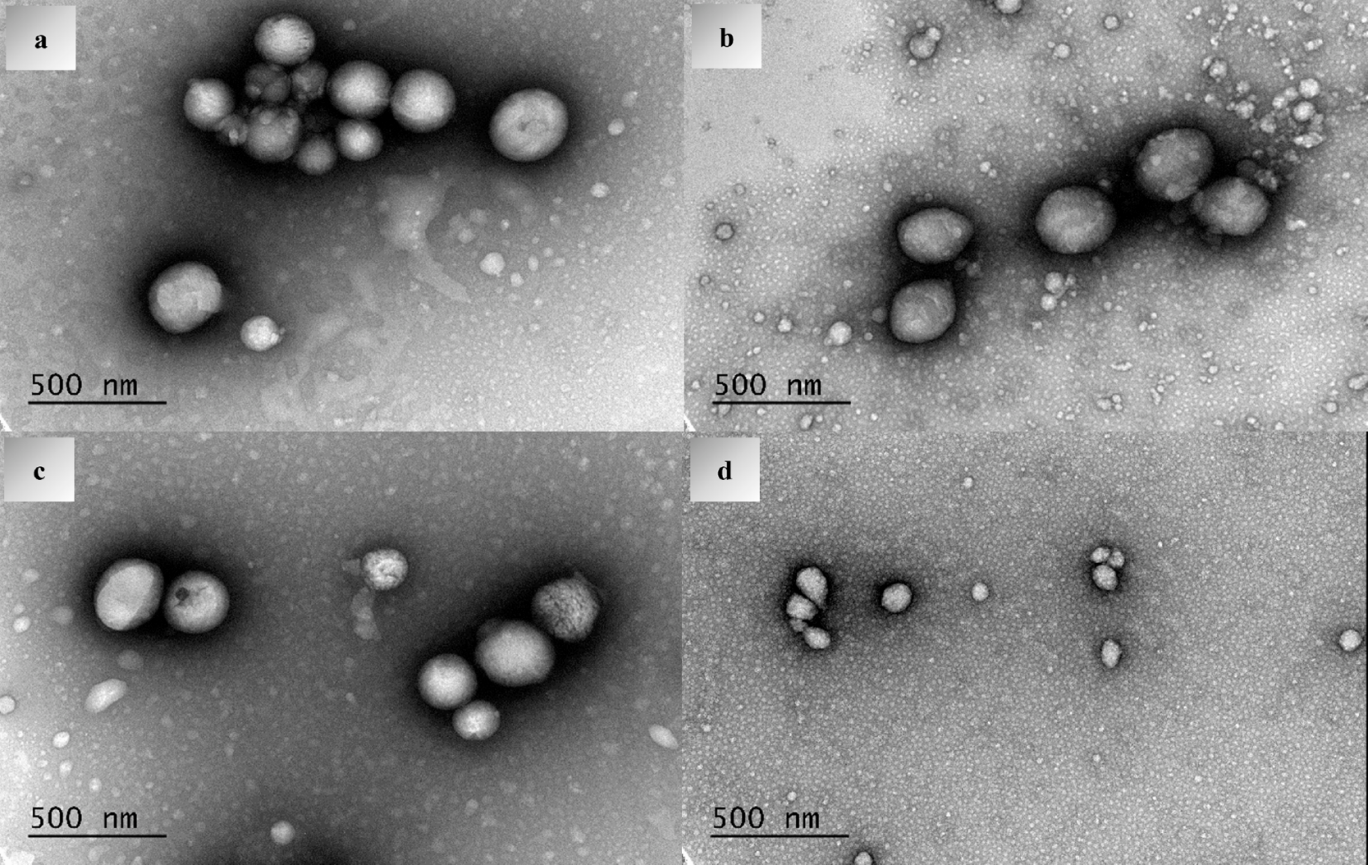
TEM pictures of (a) LN-*g*-PCL_p_ 22 DP, (b) LN-*g*-PCL_p_ 57 DP, (c) LN-*g*-PCL_p_ 101 DP, and (d) LN-*g*-PCL_a_ 103 DP nanoparticles.

The morphology of LNPs synthesized from the LN-*g*-PCL copolymer was investigated through TEM analysis. As shown in Figure 6, the LN-*g*-PCL NPs displayed regular spherical shapes within the 80–270 nm size range. The TEM results revealed distinct dark and clear regions, where the dark portions are attributed to the LN block and the clear areas correspond to the hydrophobic PCL blocks. This core–shell morphology of LNPs indicates the formation of micelle-like nanoparticles in aqueous solutions, consistent with findings from previous studies.^7^ It is important to note that the TEM analysis was performed after the drying and resuspension of the particles. Postdrying DLS analysis of particles revealed higher PDI values, aligned with the TEM observations.

### Agrochemical Loading and Entrapment Efficiency

Table 2 provides details on the loading capacity (LC) and entrapment efficiency (EE) of the agrochemical MFZ in the LNPs. LC and EE were not significantly affected by the DP of polymers made by ROP. However, lower LC and EE were presented by NPs synthesized from LN-*g*-PCL_a_ made by the acylation reaction grafting method. The reduced LC and EE of the LN-*g*-PCL_a_ NPs could be attributed to the small size of the nanoparticles and the molecular structure of the amphiphilic polymer. For LN-*g*-PCL_a_, PCL is attached at both ends, leading to a more compact and entangled PCL chain structure;^25^ hence, a smaller size nanoparticle core with lower EE was formed. On the other hand, in LN-*g*-PCL_p_, PCL is grafted from one side only, resulting in a longer chain for the same DP. The longer PCL chain structure resulted in bigger core size in a core–shell structure, resulting in higher EE. In summary, the observed differences in LC and EE emphasize the impact of the grafting method and NPs size on the entrapment properties of the NPs.

### Agrochemical Release

**Figure 7:**
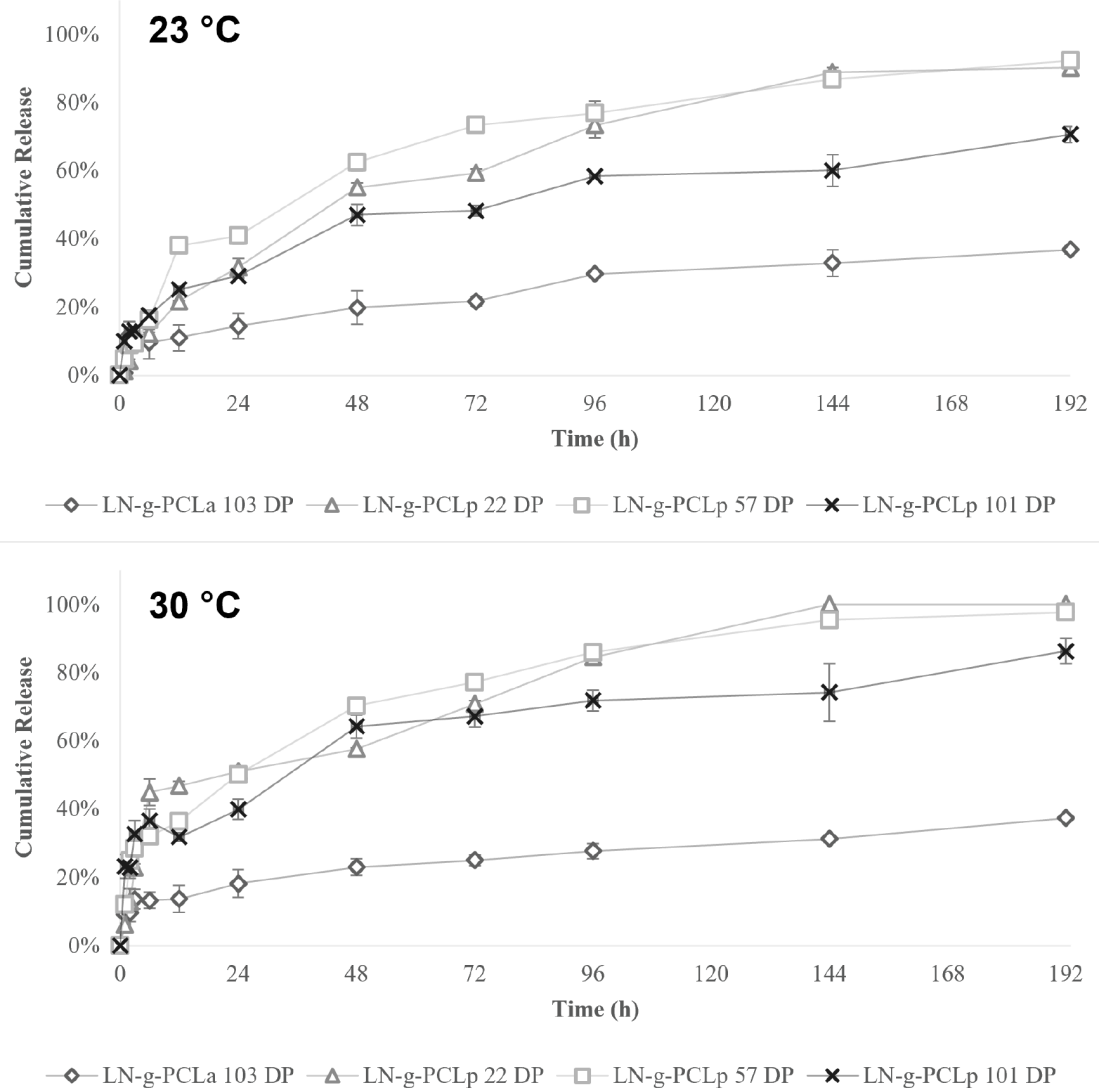
Release profile of MFZ at 23 °C (top) and 30 °C (bottom) from LN-*g*-PCL_p_ (22, 57, and 101 DP) and LN-*g*-PCL_a_ (103 DP) nanoparticles (*n* = 3).

The agrochemical release profile from LN-*g*-PCL NPs was investigated, considering variations in the DP, grafting methods, and temperature increase from 23 to 30 °C. Among NPs with different DP achieved by the ROP grafting process, the LN-*g*-PCL_p_ 22 DP, with the shortest PCL chains attached to LN, released 31.67% of MFZ in the first 24 h and behaved similarly to LNPs from the LN-*g*-PCL_p_ 57 DP, while LN-*g*-PCL_p_ 101 DP NPs showed a lower cumulative release of 29.19% of MFZ during the same time (Figure 7). These results suggest an influence of DP on the cumulative release. Grafting methods also influenced the release of MFZ from the LNPs. LN-*g*-PCL_a_ NPs, containing PCL chains with a DP of 103 and a smaller size, showed the lowest cumulative release of MFZ compared with all other LNPs.

The slower release from LN-*g*-PCL_a_ relative to LN-*g*-PCL_p_ can be attributed to the fact that the two grafting methods result in different chemical structures of the polymers, leading to variations in the internal structure of the NPs, and this variation impacts the diffusion of MFZ. The small and compact PCL core matrix in LN-*g*-PCL_a_ NPs presented more restricted PCL chains and slower diffusive motion due to the relaxation of grafted chains.^25,29^ The more densely packed and entangled PCL core could reduce the diffusion of MFZ though the polymeric matrix, resulting in a higher binding affinity between MFZ and the PCL core of the nanoparticle influencing the MFZ release profile.

Previous studies indicated that increasing the hydrophobic core of the nanoparticles, achieved with increasing DP of PCL, can delay the release of hydrophobic agrochemicals.^33^ While no discernible differences were found in the cumulative release over the initial hours, possibly due to release of weakly bound MFZ located near the shell at core–shell NPs structure, after 24 h, the strong interactions of PCL with MFZ bound in the core of the NPs, due to its hydrophobic nature, lead to a gradual, DP-dependent release. After the first day, the slow release of MFZ from NPs may be related to the presence of MFZ in the core, where MFZ is protected from hydrolysis due to the hydrophobic nature of PCL.^28,34^ The increase in DP of PCL grafted to LN molecules increases the chance of entanglement between PCL–PCL chains during the NPs formation, influencing the structure and stability of the micelles formed.^29^ This increased entanglement decreases the accessibility of water molecules to the core and the diffusivity of MFZ through the PCL matrix, resulting in slower release for higher DP of PCL in LN-*g*-PCL NPs. These results also align with the no increase of NPs size between 55 and 101 DP during micelle self-assembly.

Temperature influenced the release behavior, where a faster release was expected at higher temperatures due to the accelerated diffusion of MFZ from the polymeric matrix into the media, as noticed in previous studies.^10^Figure 7 revealed no notable differences between the release profile of LN-*g*-PCL_p_ NPs with 22 DP (100%) and 57 DP (97.69%) over 196 h at 30 °C, whereas LN-*g*-PCL_p_ 101 DP NPs showed a more delayed release (86.28%) compared to lower DP. All LN-*g*-PCL_p_ NPs increased the total cumulative release over 196 h h when the temperature was 30 °C. In contrast, LN-*g*-PCL_a_ NPs demonstrated the lower cumulative release of all four polymeric NPs (37.31%), which was not significantly affected by the temperature increase (36.78% at 23 °C).

Overall, these findings contribute to our understanding of parameters that can be manipulated to modulate the MFZ release from LN-*g*-PCL NPs as delivery systems for the controlled release of agrochemicals, with potential applications in agricultural practices for insect control.

## Conclusion

In summary, LN was modified by two grafting methods, “grafting to” acylation reaction and “grafting from” ROP, and two different DP of PCL attached to LN using the ROP method were achieved. The resulting amphiphilic LN-*g*-PCL copolymers demonstrated self-assembly into NPs, enabling the entrapment and controlled release of insecticide MFZ. FTIR confirmed the attachment of PCL to LN, while NMR results showed different DP on copolymers and changes in the OH content of grafted LN.

The LNPs presented a spherical shape, and their average sizes were affected by the grafting methods; LNPs size ranged from 80.21 ± 0.2 nm (acylation) to 184.6 ± 3.5 nm (ROP). Additionally, the increase in DP in ROP synthesized polymers led to an increase to 245.0 ± 1.5 nm. Grafting methodologies, the DP of LN-*g*-PCL_p_, and temperature variations during release studies all affected release profiles. LNPs from the acylation reaction grafting method showed a cumulative release of MFZ of only 36.78 ± 1.23%, over 192 h, while an increase in DP through ROP grafting method reduced cumulative release from 92.39 ± 1.46% to 70.59 ± 2.40% over the same time frame. In general, this study demonstrated the potential of LNPs to control the release of agrochemicals using renewable materials, leading to sustainable agricultural applications. Further research should focus on developing new nanostructures employing biodegradable copolymers to address plastic waste concerns in agriculture.

## Data Availability

The text generated by ChatGPT-3.5 in response to the prompts are available from the corresponding author upon request.

## Supporting Information Available

Prompts used to refine the text with aid from ChatGPT-3.5; size distribution histograms by intensity of (a) LN-*g*-PCLp 22 DP, (b) LN-*g*-PCLp 57 DP, (c) LN-*g*-PCLp 101 DP, and (d) LN-*g*-PCLa 103 DP nanoparticles (PDF)

## Abbreviations

C=O: carbonyl group
CDCl_3_: deuterated chloroform
CH_2_: methylene
CL: ε-caprolactone
DCM: dichloromethane
DLS: dynamic light scattering
DMF: dimethylformamide
DMSO: dimethyl sulfoxide
DP: degree of polymerization
DSC: differential scanning calorimetry
EE: entrapment efficiency
FDA: Food and Drug Administration
FT IR: Fourier transform infrared spectroscopy
HPLC: high-performance liquid chromatography
LC: loading capacity
LN: lignin
LN-*g*-PCL: lignin-grafted-poly(ε-caprolactone)
LN-*g*-PCL_a_: LN-*g*-PCL by acylation reaction
LN-*g*-PCL_p_: LN-*g*-PCL by ROP
LN-*g*-PLGA: lignin-grafted-poly(lactic-*co*-glycolic) acid
LNPs: lignin nanoparticles
MFZ: methoxyfenozide
MRDT: maximum rate of degradation temperature
NMR: nuclear magnetic resonance
NPs: nanoparticles
OH: hydroxyl group
PCL: poly(ε-caprolactone)
PLGA: poly(lactic-*co*-glycolic) acid
ROP: ring-opening polymerization
Sn(Oct)_2_: stannous 2-ethyl hexanoate
*T*_c_: crystallization temperature
TEM: transmission electron microscopy
*T*_g_: glass transition temperature
TGA: thermogravimetric analyses
*T*_m_: melting temperature
TMDP: 2-chloro–4,4,5,5-tetramethyl-1,3,2-dioxaphospholane
*X*_c_ (%): crystallinity.
