# Supplementary material for: Modulation of Methoxyfenozide Release from Lignin Nanoparticles Made of Lignin Grafted with PCL by ROP and Acylation Grafting Methods
Source: Langmuir. 2024 Mar 1;40(10):5433–43. doi: 10.1021/acs.langmuir.3c03965 (PMC10938892; doi:10.1021/acs.langmuir.3c03965)
Supplement: Supplementary file 1 — la3c03965_si_001.pdf [file la3c03965_si_001.pdf]

Supporting Information

Modulation of Methoxyfenozide Release from Lignin Nanoparticles Made of Lignin Grafted with PCL by ROP and Acylation Grafting Methods

Alvaro Garcia[1], Carlos E. Astete[1], Rafael Cueto[2], Cristina M. Sabliov[1]*

[1] Biological & Agricultural Engineering, Louisiana State University and LSU Ag Center, Baton Rouge, Louisiana 70803, United States

[2] Department of Chemistry, Louisiana State University, Baton Rouge, Louisiana 70803, United States

*Corresponding author, csabliov@lsu.edu

Table of Contents

S1. Prompts used to refine the text with aid from ChatGPT-3.5. .... S2

Figure S1. Size distribution histograms by intensity of (a) LN-g-PCLp 22 DP, (b) LN-g-PCLp 57 DP, (c) LN-g-PCLp 101 DP, and (d) LN-g-PCLa 103 DP nanoparticles................................ S3

S1. Prompts used to refine the text with aid from ChatGPT-3.5.

Evaluate the clarity and transition words of the following introduction for a scientific journal in ACS Journal and give suggestions.

Give suggestions for transition sentences or words, and examples about how to use it.

Evaluate the following text from a result and discussion section for a scientific journal for clarity.

Evaluate the following text for a conclusion section for a scientific journal for clarity.

Evaluate the following text for an abstract for a scientific journal in ACS journal for clarity.

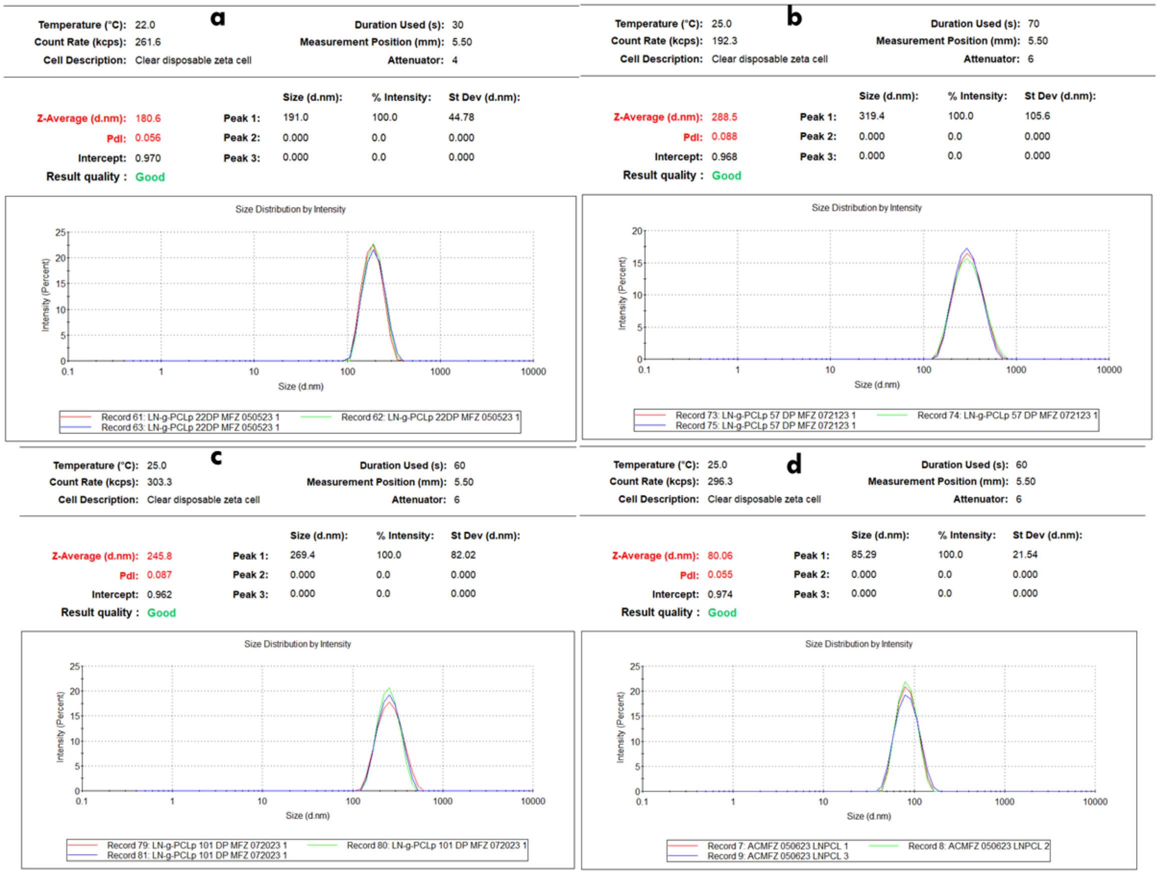

Figure S1. Size distribution histograms by intensity of (a) LN-g-PCLp 22 DP, (b) LN-g-PCLp 57 DP, (c) LN-g-PCLp 101 DP, and (d) LN-g-PCLa 103 DP nanoparticles. These measurements were done before drying the NPs using a freeze-dryer.
